# Adeno-Associated Virus (AAV) Versus Immune Response

**DOI:** 10.3390/v11020102

**Published:** 2019-01-25

**Authors:** Joseph Rabinowitz, Ying Kai Chan, Richard Jude Samulski

**Affiliations:** 1 Senior Director of Capsid Development, Rare Disease Research Unit, Pfizer Inc., 7030 Kit Creek Road, Morrisville, NC 27560, USA; 2 Department of Genetics, Harvard Medical School, Boston, MA 02115, USA; 3 Wyss Institute for Biologically Inspired Engineering, Harvard University, Boston, MA 02115, USA; 4 Department of Pharmacology, School of Medicine, University of North Carolina at Chapel Hill, Chapel Hill, NC 27599, USA

**Keywords:** rAAV, immune response, rare diseases

## Abstract

Decades ago, Friedmann and Roblin postulated several barriers to gene therapy, including tissue targeting, delivery across the blood–brain barrier (BBB), and host immune responses. These issues remain pertinent till today. Since then, several advances have been made in elucidating structures of adeno-associated virus (AAV) serotypes, antibody epitopes, and ways to modify antibody-binding sites. AAVs capsid has also been engineered to re-direct tissue tropism, reduce ubiquitination, and promote passage across the BBB. Furthermore, the use of high(er) dose recombinant AAV (rAAV) has been accompanied by a better understanding of immune responses in both experimental animals and early clinical trials, and novel work is being performed to modulate the immune response. While the immune responses to rAAV remains a major challenge in translating experimental drugs to approved medicine, and will likely require more than a single solution, we now better understand the hurdles to formulate and test experimental solutions to surmount them.

## 1. Introduction

Friedmann and Roblin postulated several barriers to gene therapy decade ago [1]. Since then, adeno-associated virus (AAV) has emerged as a promising gene therapy vector and its use is being explored for numerous therapeutic applications. Wild-type AAVs are non-replication competent parvoviruses (dependovirus) that require a helper virus or cellular stress to replicate. They were discovered in the 1960s as contaminants of Adenovirus electron micrographs and were thought to be satellite viruses. These single-stranded DNA, non-enveloped viruses are approximately 24–26 nm in diameter. The virions are composed of 60 capsid subunits and they package a 4.7 kb genome that contains a 145-nucleotide inverted terminal repeat (ITR) at each end. The ITRs are secondary structure elements protecting the linear single-stranded genomes that have fewer than 10 unpaired nucleotides, outside the D-sequence, and contain regions required for AAV replication and packaging. Between the ITRs are the replication (Rep), capsid (Cap, comprising Vp1, Vp2, and Vp3), and assembly-activating protein (AAP) open reading frames.

There are four non-structural Rep proteins (Rep78, 68, 52, and 40). The differences in their names refer to their molecular weights due to p5 (rep 78 and 68) or p19 (rep52 and 40) promoter usage, as well as the use of an intron (Rep 68 and 40). Co-infection with a helper virus (e.g., Adenovirus) results in activation of the AAV p5 (Rep 78/68) and p19 (Rep52/40) promoters. Once p5 and p19 are activated, replication occurs via unidirectional strand displacement. The Rep proteins contain multiple functional domains, present along the protein sequence. Rep’s amino-terminal domain includes regions required for DNA binding and endonuclease activity, whereas the carboxy terminal contains a Zn finger domain, implicated in interacting with host cell factors. The central domain contains regions necessary for ATPase and helicase activity. The helicase region contains three Walker motifs that function as packaging motors and form a structural hexamer [2]. The large Rep proteins bind ITRs, promoters, as well as single strand DNA, whereas the small Rep proteins have ATP dependent helicase activity and package DNA into preformed virions, among other functions specific for the wild-type genome.

The three overlapping Cap proteins consist of Vp1 (87 kD), Vp2 (72 kD), and Vp3 (61 kD), and these proteins are transcribed using the p40 promoter, generating two transcripts (one for Vp1 and one for Vp2), using a canonical start codon for Vp3 and a non-efficient start codon for Vp2. More than 530 carboxyl terminal amino acid residues (AAs) overlap among the Cap proteins, with Vp1 and Vp2 sharing more than 60 overlapping AA, and Vp1 possessing a 137-AA unique N-terminus that contains a parvovirus genus-conserved phospholipase A2 domain required for endosome escape and efficient transduction of infected cells [3]. The virion consists of 60 capsid subunits that form T = 1 icosahedral-symmetry composed of Vp1, Vp2, and Vp3 at a 1:1:10 ratio, respectively [4]. The topology of subunits of AAV and other parvoviruses, generated from X-ray crystallography or electron cryo-microscopy, show many similar structural motifs, including eight stranded antiparallel β-sheets, βBIDG and βCHEF, that form the jellyroll motif, a large complex loop structure between beta sheets G and H, a depression at the two-fold axis, and a pore at the five-fold axis [4,5,6,7,8,9,10]. Connecting the β-sheets are loops that map, in turn, to other structural elements upon the virion. For example, the five-fold axis is composed of loop domains that lay between β-sheets H and I, as well as D and E. In contrast to the β-sheet elements, the loop elements between the β-sheets are the most variable between serotypes. The structural elements of AAV serotypes have demonstrated a plasticity that enables significant modification to be engineered upon them. This particular feature and the nearly limitless AA sequence space will allow for many traits to be grafted onto the surface of the virion.

In the future, we believe AAV DNA delivery systems will: (i) enter the body systemically; (ii) target the affected organ(s) specifically and; (iii) express the therapeutic transgene, suppression system (such as RNAi) or novel cassettes (such as gene editing approaches and ophthogenetics) at the appropriate level to mitigate a monogenic condition or of a long-term lifestyle choice. We envision AAV gene therapy would accomplish these without: (i) inducing innate or adaptive immune responses; (ii) expressing in non-target tissues or; (iii) adversely altering the normal transcriptome in the target tissue. In the last decade the science of gene therapy has taught us that barriers exist in the treatment of many “simple” gene replacement strategies, and this understanding has fueled a new vein of research that is mounting those and other barriers. In this review we will discuss new lines of research that will allow AAV gene therapy to overcome barriers of innate immune response, adaptive immune response against the transgene and vector, and targeting organ(s) systems of interest. The multi-faceted research clearly demonstrates the adaptability of the AAV gene delivery system.

## 2. The Immune Response

### 2.1. Host Response to AAV

#### 2.1.1. The Innate Immune Response

**Figure 1:**
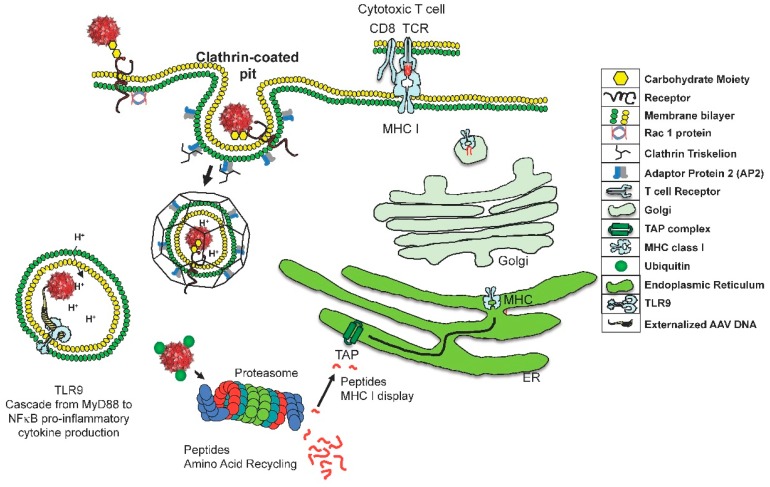
This cartoon describes two risks associated with adeno-associated virus (AAV) entrance in to a target cell. The innate immune repsonse is activated by TLR9 recognition of the externalization of the viral genome. This event is rapid and NF-kB will be activated resulting in proiinflammatory cytokine production. For the many virions that do not rapidly uncoat, a second risk exists—the slow degradation of the capsid. Proteasomes degradation of ubiquitinated AAV results in a small amount of capsid fragments being transported through the transporter associated with antigen processing (TAP), into the endoplasmic reticulum (ER), and innevitable attachment to major histocompatibility complex (MHC) class I molecules. These peptides will be displayed on the cell surface and eventually recognized by cytotoxic T cells.

Toll like receptors (TLRs) are a family of pattern recognition receptors (PRR) present in vertebrates and invertebrates. The human and mouse family of TLRs contain 13 members (TLR1-13). Each TLR is a single pass transmembrane receptor with TLR1, 2, 4, 5, 6, and 10 on the plasma membrane and TLR3, 7, 8 and 9 in the endosome [11]. All TLRs play a fundamental role in the activation of innate immunity by detecting pathogens (via pathogen associated molecular patterns [PAMPs]) and cell damage (via damage associated molecular patterns [DAMPs]), and trigger a signaling cascade that leads to innate immune response activation. With the exception of TLR3, all other mammalian TLRs signal through the signaling adaptor myeloid differentiation primary response gene 88 (MyD88), and this cascade leads to phosphorylation and degradation of Inhibitor of NF kappa B (IκB), thereby releasing inhibition on NF-κB and allowing it to translocate to the nucleus to activate transcription of several pro-inflammatory genes [12]. TLR9, present on the endosomal membranes of immune cells, binds to a specific unmethylated DNA sequence, the CpG motif (cytosine-phosphate-guanine), present in the genomes of bacteria and DNA viruses. For example, DNA viruses such as Adenoviruses may uncoat and release their genomes in the endosome, allowing TLR9 binding and activation of the innate immune response. During AAV endosomal trafficking, virion uncoating and/or partial exposure of the genome may result in TLR9 sensing of CpG motifs. Zhu et al. revealed that innate immune recognition of AAV2 by mouse or human plasmacytoid dendritic cells (pDCs) was mediated by TLR9, dependent on MyD88 signaling, and was independent of the form of transgene or capsid serotype [13]. The authors also found that the TLR9-MyD88 pathway was critical for CD8+ T cell responses to AAV2 capsid and the transgene product in mice following intramuscular injection [13]. Activation of TLR9 by AAV genomes has also been shown to be dependent on CpG dinucleotides in vivo. First, a study compared expression in wild-type (wt) control and TLR9 knockout mice, and showed that transgene expression was eliminated in wt but not in TLR9 knockout animals after delivery by AAVrh32.33 [14]. AAVrh32.33 injection in mice results in a robust T cell response to both the capsid and the transgene product, this is unlike the T cell response to other AAV serotypes in mice [15]. In a second experiment, the transgene either contained (308) or was depleted of CpG dinucleotides (zero), and AAVrh32.33 was used for delivery into skeletal muscle of wt mice. The wt transgene lost expression over 30 days while the CpG depleted transgene-maintained expression for >90 days [14] (Figure 1). In mice, a difference in AAV genome configuration may influence the innate response; self-complimentary AAV genomes increased the innate immune response to transgene, whereas the response to single stranded vectors was less severe [16]. Finally, a study reported that primary human liver cells can mount an innate immune response against AAV2 and AAV8 capsid in vitro via TLR2 [17]. These experiments demonstrate that both the DNA genome and the protein capsid can influence the activation of the innate immune response, which is critical for shaping the adaptive immune response.

#### 2.1.2. Host Response Towards AAV Capsid

Recombinant AAV was initially thought to be minimally immunogenic, an attribute of the vector’s ability to achieve sustained transgene expression in some experimental animal models. However, natural infection studies demonstrated existing wt AAV in human samples, and the presence of neutralizing antibodies in healthy subjects throughout the world [18,19] or in specific gene therapy target populations [20]. The level of pre-existing antibodies is high enough to reduce the inclusion population of patients for these rare diseases by as much as 50% [18,19,20,21]. Therefore, it is not surprising that efficacy of gene transfer has been compromised in several early clinical trials due to attendant immune responses. In the earliest AAV2 factor IX clinical trials, transient transgene expression was seen for up to eight weeks before loss in a subset of patients. Subsequently, it was determined to be an AAV capsid-specific CD8+ cytotoxic T cell response that likely led to the decline of factor IX expression, while other patients had preexisting humoral antibodies [22].

After transduction, some AAV virions are ubiquitinated and degraded within proteasomes, and their peptides processed through the transporter associated with antigen processing (TAP) [23]. This processing allows capsid peptide fragments to be bound to the MHC class I molecule, which are transported to the cell surface for display, resulting in destruction of transduced cells by cytotoxic T cells. Proteasome inhibitors have been shown to increase transduction in vitro and reduce the presentation of capsid peptide fragments on MHC class I molecules [24,25]. In addition, a reduction in serine and threonine AAs on the virion has led to increased transduction in vitro and in vivo in a serotype independent manner [26,27]. This suggests that processes to reduce ubiquitination and proteasome degradation of the virion act together to reduce display of virion peptides on MHC class I molecules. Hui et al [28] performed a quite detailed study of the human immune response to AAV1 and AAV2 capsids in splenocytes isolated from human spleens. In unstimulated splenocytes, few of the 44 subjects’ splenocytes demonstrated a T cell response. However, after restimulation with AAV peptides, 20 of 32 patient splenocytes were positive in IFN-γ ELISPOT assay [28]. Of particular interest, only a subset of the 140+ peptides used to stimulate the splenocytes were immunodominant. In a subsequent trial for hemophilia B using AAV8, individuals were excluded from participation due to the presence of pre-existing antibodies against the specific serotype capsid, and no immune response was detected against the transgene product. However, capsid-specific T cell responses were detected and coincided with increased aspartate aminotransferase and alanine aminotransferase values, and concomitant decline in plasma factor IX levels. These patients were treated with steroids and the high dose group maintained adequate factor IX levels [29]. At the 2017 ASGCT annual meeting, Spark Therapeutics reported on a trial where patients have sustained expression of approximately 30% wild-type levels of factor IX using the Padua variant (NCT02484902), an R338L mutation that significantly increases the activity of factor IX. The loss of factor IX expression in these patients without neutralizing antibodies parallels the loss seen in mouse models that examined the immune response to transgene product.

#### 2.1.3. Host Response towards AAV Transgene Product

Patients who have null mutations in genes do not express any protein and; therefore, lack self-tolerance against the gene product. These patients present the most pressing immune challenge due to the need to induce tolerance to the “foreign” therapeutic protein. While deletions and frame shifts in the Duchenne’s muscular dystrophy (DMD) gene may allow some of the dystrophin gene to be expressed, other domains of the protein may be seen as foreign. This was the case in an AAV DMD clinical trial, in which patient 5 developed CD8+ T cells against a deleted region of the endogenous mutant dystrophin that was present on the therapeutic protein [30]. Displaying fragments of the transgene product on the surface of antigen-presenting cells or cytotoxic T lymphocytes is a major barrier towards successful gene delivery. In a recent manuscript, varying the dose of AAV8 encoding ovalbumin (OVA) resulted in a counter intuitive induction of the immune response in a mouse model. At the middle dose (1 × 10^9^ vg/mouse) there was a two-month delay in CD8+ T cell transgene response that corresponded to a loss of surface expression of programmed death 1 (PD-1) on specific systemic CD8+ T cells [31], while at the low (1 × 10^8^ vg) and high dose (1 × 10^10^ vg) there was no systemic CD8+ T cell response and only a liver-localized response at the low dose. In the middle dose group, expression of inhibitory molecules on CD8+ T cells was examined and both 2B4 and PD-1 expression were reduced, followed by loss of transgene expression [31]. In the skeletal muscle there may be additional responses that lead to immune activation. How can this adaptive response be ameliorated?

### 2.2. Adaptive Immune Response

Phase I/II clinical trials have provided insight into the existing challenge of adaptive immune responses on the safety and efficacy of AAV gene transfer. In a phase I clinical trial for the treatment of Hemophila B (F.IX) using AAV2, transgene expression transiently peaked at five-weeks post-delivery for the highest dose patient, followed by progressive loss of F.IX expression. Additional studies implicated a capsid-specific CD8^+^ T cell population that recognized and destroyed AAV-transduced cells, coincident with the presence of activated PBMCs against epitopes on the capsid [22]. This patient initially exhibited a therapeutic level of factor IX that subsequently declined. Similarly, patients affected with limb girdle muscular dystrophy (LGMD) who received AAV1 intramuscularly expressing an γ-sarcoglycan transgene, or DMD patients that received an AAV vector expressing the mini-dystrophin transgene, exhibited immunity toward vector antigens and demonstrated limited transgene expression [30]. Consequently, transient immunosuppression, to combat immunity toward AAV capsid antigens following gene delivery, has been adopted as the therapy of choice and functions as a “safety net”. A three-day course of methylprednisolone beginning 4 prior to gene transfer injection was administered to patients participating in a clinical trial for LGMD [32]. Both cellular and humoral responses toward AAV1 were detected in one patient. Six months post administration, two out of three patients expressed the delivered transgene, while the third patient exhibited detectable humoral and cellular immunity and showed no expression [33]. In another DMD trial, cellular immune responses to either self- or non-self-dystrophin epitopes were detected in four out of six patients, with all six failing to express the delivered mini-dystrophin protein in myofibers at two biopsy time points [30]. Immunosuppressants, including glucocorticoids such as prednisolone, were administered. These results suggest that mechanistic studies based on new vector design strategies that circumvent the induction of the innate and adaptive immune response toward AAV associated antigens are essential to improve safety and efficacy of AAV gene transfer in clinical settings.

### 2.3. Approaches to Modulating the Antibody Response to AAV

#### 2.3.1. Bioengineering of AAV Virions

Much has been written concerning the mutagenesis of AAV capsids. Girod et al. [34] used guided insertional mutagenesis of AAV2 to insert a 14-AA peptide into six-putative loop regions, using the canine parvovirus crystal structure as a guide. Rabinowitz et al. [35] used random insertional mutagenesis, placing four to five AAs into 22 sites, spanning all three capsid regions. These early mutagenesis studies demonstrated that virions were flexible to substitutions and insertion as well as defining AAs as important to known viral functions (e.g., heparin binding). After the crystal structure of AAV was determined, researchers using mutagenesis had a structural blueprint [4]. Lochrie took advantage of this blueprint to examine mutations in 64 positions that were all surface displayed [36]. Screening these mutations against the A20 monoclonal antibody (a monoclonal antibody that binds the intact virion) and pooled human Immunoglobulin G (IgG) revealed positions that, when mutated, reduced binding and neutralization by A20 (Q263, D264, S384, Q385, E548, and V708), and either binding or neutralization by pooled sera [36]. Furthermore, the 21 positions were dispersed over a large area on the virion surface. However, it was not until the adoption of DNA shuffling, and DNA family shuffling combined with error prone PCR [37], that mutagenesis of AAV capsids increased the diversity by orders of magnitude when compared to naturally occurring serotypes [38,39,40]. Using this method, fragmented capsid genes are reassembled in a self-primed polymerase reaction, where multiple serotype templates are joined at regions of sequence homology. Coupling this reassembly with error prone PCR further increased the diversity of resulting chimeric capsids. This is a form of molecular breeding that results in large capsid pools that can be examined for functional diversity. The functionality can be examined by providing a selective force upon the pool.

#### 2.3.2. Engineering Immune Response Avoidance by Directed Mutagenesis

The ubiquitin-proteasome system is a cellular pathway leading to degradation of proteins at the proteasome complex. Studies dating back more than two decades have shown that phosphorylation of AAs is one of the signals that promote binding of ubiquitin ligases (E3). In the case of I Kappa B degradation, serine phosphorylation is the key modification leading to ubiquitination [41]. Proteasomes degrade ubiquitinated proteins into small peptide fragments that are, for the most part, too small to be taken into the endoplasmic reticulum (ER) by the transporter associated with antigen processing (TAP) [42]. To bind, MHC class I peptides must be between eight and 10 AAs in size; once bound to MHC class I they are transported to the cell surface to display the bound peptide (Figure 1).

In a logical series of experiments [26,43], Zhong et al. demonstrated that AAV2 interaction with epidermal growth factor receptor protein tyrosine kinase (EGFR-PTK) inhibited transduction and targeted AAV2 for degradation by the proteasome, and by inhibiting EGFR-PTK there was an increase in transduction efficiency in vitro. Subsequently, the mutated surface displayed tyrosine (Y) residues to phenylalanine (F), and some mutants increased transduction efficiency in vitro and in vivo [43]. These mutations were extended to other targets of phosphorylation (serine, threonine, and lysine residues) and to other serotypes (AAV6 and AAV8), and examined in large animal models [27,44]. In many cases the results of the in vitro experiments were recapitulated in vivo [45], suggesting ubiquitination is a conserved (ancient) process. One of the byproducts of reducing proteasome degradation is limiting the display of peptides on MHC class I molecules. This suggests other proteases are involved in AAV degradation. In a two-hybrid screen, cathepsins B and L were shown to interact with the virion in the endosome, potentially inducing degradation [46]. These experiments demonstrate how an understanding of cellular degradation was used to modify the virion leading to increased transduction efficiency. In the next section a detailed understanding of antibody binding is used to design modifications of the virion that can potentially reduce the neutralizing antibody exclusion criteria in the treatment of disease populations.

#### 2.3.3. CryoEM Structure-Guided Mutagenesis to Reduce Neutralizing Antibody Binding

Researchers have elucidated AAV crystal structures starting with AAV2 [4] and followed shortly by additional serotypes [6,7,8,9]. More recently the use of cryoelectron microscopy (CryoEM) has resulted in resolutions comparable to crystallography, with the benefit of speed, small sample size, and the ability to visualize protein binding interactions between AAV serotypes and antibodies. It is noteworthy that CryoEM is experiencing a renaissance, including the topic of the 2017 Nobel Prize in Chemistry (nobelprize.org). Early attempts to map neutralizing antibodies complexed to parvovirus capsids by CryoEM were successful at mapping antigenic AAs, with the help of neutralization escape mutants for verification [47]. With increasing resolution, AAV serotypes complexed with fragment antigen binding (Fab) domains of neutralizing MAbs have been imaged by CryoEM and AA residue footprints determined [48,49,50]. Antibodies have two Fabs, one at the end of the Y and a constant domain at the other end. The distance between two Fabs of an antibody is roughly 15nm and this distance may preclude binding of both Fabs to one virion. Additionally, there are differences between Fab-footprints (occluded) residues and the contact residues, and CryoEM can now determine the contact residues. For example, the AAV1 binding footprint of MAb 4E4 contains residues from variable regions (VR) III–IX, occluding 83 residues, but contacts residues 456-AQNK-459 and 492-TKDNNN-498 from VR IV and V, respectively [48]. Interestingly, Lochrie identified R471A as a point mutation of the capsid surface that increased the resistance to human sera neutralization [36]. For the majority of the AAV CryoEM with mapped antigenic residues, the footprints are in the variable domains proximal to the three-fold axis of symmetry, this includes AAV1, AAV2, and AAV8. However, for AAV5 the antigenic footprint flanks the 2/5-fold wall [49,50]. CryoEM antigenic footprint mappings combined with directed evolution of antigenic footprint AAs were used to alter the regions surrounding the three-fold axis that had been mapped by CryoEM as antigenic [51]. The utility of this combination of approaches is demonstrated by the maintenance of receptor binding, transduction, and packaging efficiency when directed evolution was performed on these regions. CryoEM, as an analytic tool, has both speed and resolution, and in the future it may be possible to bind patient Fab domains to therapeutic virions and directly examine binding properties.

#### 2.3.4. Engineering Antibody Evasion by Directed Evolution

There is a significant amount of power in disorder. Taking advantage of error in replication fidelity coupled with random assembly of similar but divergent DNA fragments, at regions of localized homology, results in accelerated directed evolution [37]. Applying this strategy, methods have been developed to introduce mutations into viral structural genes by error prone PCR, and by generating chimeric capsids by fragmentation of multiple serotype capsid genes and reassembly at overlapping regions of homology (family shuffling). After generation of virions with the variant capsid gene, within the shell it codes for, screening can begin. To engineer AAV with greater resistance to neutralizing antibodies, error prone PCR was used to alter the coding sequence of AAV2 capsid protein and to screen the pools against AAV2 mAb and pooled human sera [52,53]. Point mutations that resulted in the greatest resistance to human sera, screened for AAV neutralization ability, were R459K and N551D, resulting in 10-fold greater resistance [53], and E12A, K258N, T567S, N587I, and T716A, resulting in 100-fold greater resistance [52]. A second method to increase resistance to NAb used family shuffling of multiple AAV serotypes, where Li et al. [40] used AAV1-6, 8, and 9 to generate the chimeric vector 1829. This chimera displayed resistance against the AAV2 NAb A20 and NAb generated in mouse sera against AAV1, 8, and 9 [40]. In a second study, AAV2, 8, and 9 were shuffled to generate chimera AAV-DJ, which had a mixed resistance profile. When mice were injected with AAV2 and three weeks later injected with AAV-DJ, there was no cross-reactivity. However, when first injected with AAV8 or 9, the authors observed cross-reactivity. More recently, a group used AAV1-9 (except 7) and included Avian- and Bovine-AAV to generate a family shuffling library to explore selective human liver transduction in a xenograft liver model. The best chimera, AAV-LK03, contained AAV serotype 3B, as the component of Vp2 and Vp3, and a mixture of AAV2, 3, 4, 6, and 9, as the Vp1 unique component. When tested against two IVIG neutralization preparations, AAV-LK03 demonstrated significant levels of resistance, which is somewhat surprising considering the amount of cross-reactivity between AAV serotypes in the human population [21], and AAV2 and AAV3b in particular [20]. Finally, using cryoEM-reconstruction to identify antigenic footprints on AAV serotypes 1, 2, 5, 6, and 8, the variable regions were mutated using oligonucleotides with degenerate coding regions flanked by regions of homology. Illumina sequencing of the unselected library showed minimal bias, while the selected library showed increased lead selection [51]. By reducing the mutagenesis footprint, other important properties of virion morphology, packaging efficiency, genome titer, and transduction biases were similar to parental serotypes [51].

## 3. Conclusions/Discussion

The human immune response is multilayered and evolving. While this is important for our health, it is a robust obstacle in the development of gene therapy into a medicine. The flexibility of the AAV capsid has proven essential to generating work-a-rounds to bypass some of these obstacles. In addition, alterations in the sequence composition have also shown promise in altering the immune response. In naïve animals, or individuals with no pre-existing exposure, activation of the innate immune response is the first interaction between the individual and the viral agent. Reducing the number of CpG di-nucleotides in the viral genome inhibits activation of the innate immune response via TLR9. Modification to the capsid in the form of tyrosine to phenylalanine mutants reduces the ubiquitination of the virion. This has been shown to increase transduction and reduce the level of MHC class I peptide display. Both these strategies are passive modifications that can be employed. Individuals with pre-existing neutralizing antibodies are presently excluded from clinical trials and, in the future, they may be excluded from participating in the medical application of gene therapy. To overcome this obstacle, groups have adopted two tactics. One tactic is to use rational design based on CryoEM-antibody binding data and mutation of specific sites determined from the cryoEM data to generate escape mutants. The second tactic is directed evolution. By using large numbers of random recombinants and point mutants combined with a screen, it is possible to select a small number of mutants that escape neutralization. The screen can use specific neutralizing antibodies (e.g., A20), pooled sera, or sera from individuals with a specific disease indication. Again, these approaches are passive in that they do not require immune suppression or decoys to function. Interestingly, these approaches have narrowed the sites required for escape mutant to regions around the three-fold axis of symmetry. Overcoming the immune response may become a routine and individualized aspect of providing gene therapy to individuals with rare diseases in the near term, and it will require basic principles to be understood to provide cost effective gene medicine for larger disease indications.
